# Pyoderma Gangrenosum as a Presenting Symptom of Inflammatory Bowel Disease

**DOI:** 10.7759/cureus.91303

**Published:** 2025-08-30

**Authors:** David X Li, Tyler R Jarrett, G. Peter Sarantopoulos, Jonathan F Garcia

**Affiliations:** 1 Internal Medicine, University of California Los Angeles, Los Angeles, USA; 2 Pathology, University of California Los Angeles David Geffen School of Medicine, Los Angeles, USA; 3 Hospital Medicine, University of California Los Angeles, Los Angeles, USA

**Keywords:** crohn’s disease, ibd, inflammatory bowel disease, pyoderma, pyoderma gangrenosum

## Abstract

Pyoderma gangrenosum (PG) is a rare ulcerative dermatosis, with over half of cases associated with a systemic inflammatory condition. PG is a rare condition, and its diagnosis often proves elusive as its clinical and histopathologic findings are nonspecific; indeed, it is considered a diagnosis of exclusion. We present a case of PG as the presenting symptom of a patient’s previously undiagnosed inflammatory bowel disease, highlighting the necessity of initiating a broad work-up for associated systemic inflammatory conditions when a diagnosis of PG is suspected or confirmed.

## Introduction

Pyoderma gangrenosum (PG) is a rare ulcerative dermatosis characterized by rapidly progressive, painful ulcers, most commonly located on the lower extremities or trunk [1]. The prevalence of PG is low, estimated at between 5.6 and 6.1 cases per 100,000 adults in a cross-sectional analysis of electronic health records in the United States, with a female-to-male ratio of greater than 1.8 across all age groups [2]. Although frequently misdiagnosed as infectious ulcers, PG is inflammatory in etiology, and over half of patients with PG have an associated systemic inflammatory condition such as inflammatory bowel disease (IBD), inflammatory arthritis, or malignancy [3-5]. We present a case of PG as the presenting symptom of a patient’s previously undiagnosed IBD.

## Case presentation

**Figure 1:**
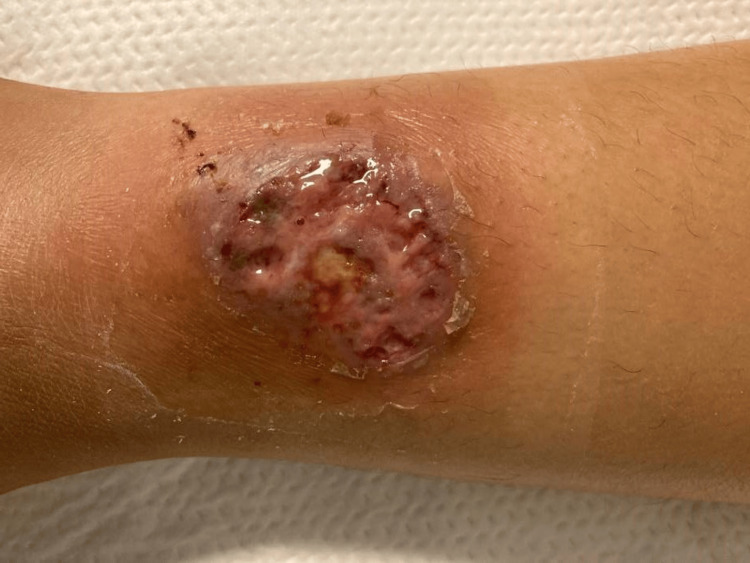
Ulcerative lesion on the left lower extremity consistent with pyoderma gangrenosum.

**Figure 2:**
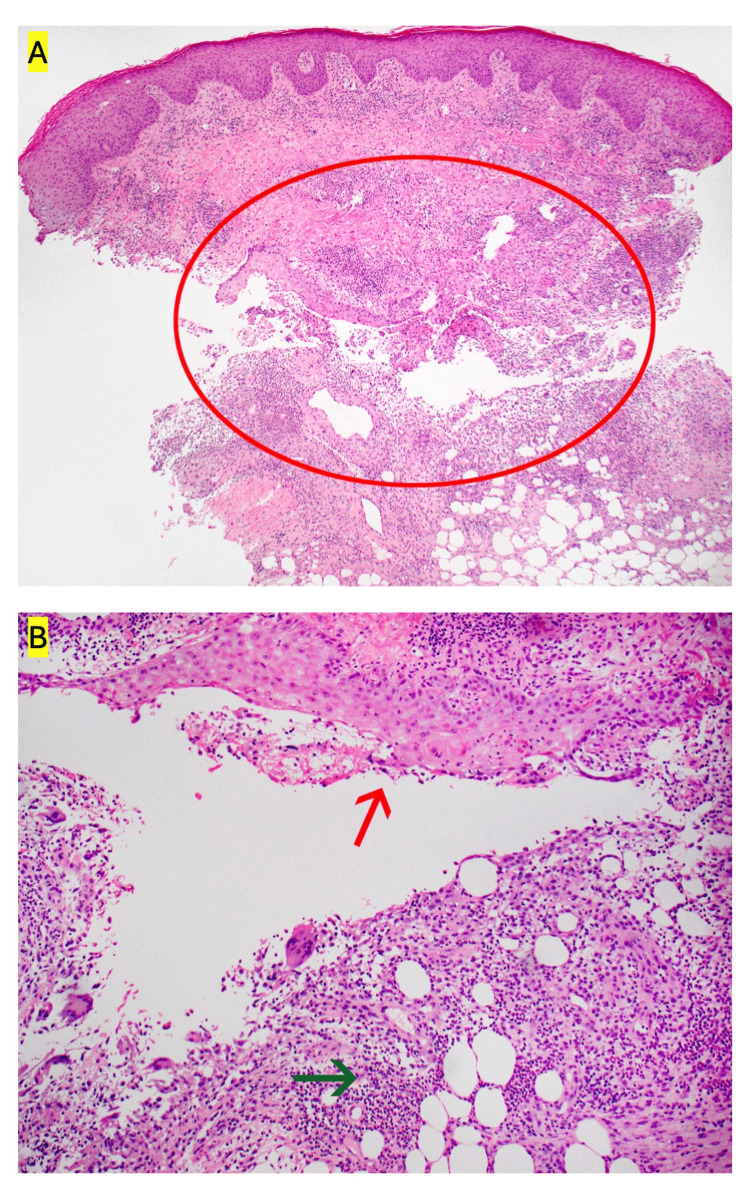
Hematoxylin and eosin stain from the skin biopsy demonstrating (A) marked mixed dermal inflammation and granulation tissue with likely sinus tract formation; a tangentially sectioned “undermined” ulcer border remained an additional consideration. (B) Higher magnification of the embedded epithelium showed markedly reactive, non-dysplastic epithelium (red arrow) with surrounding marked mixed inflammation to include numerous neutrophils (green arrow).

**Figure 3:**
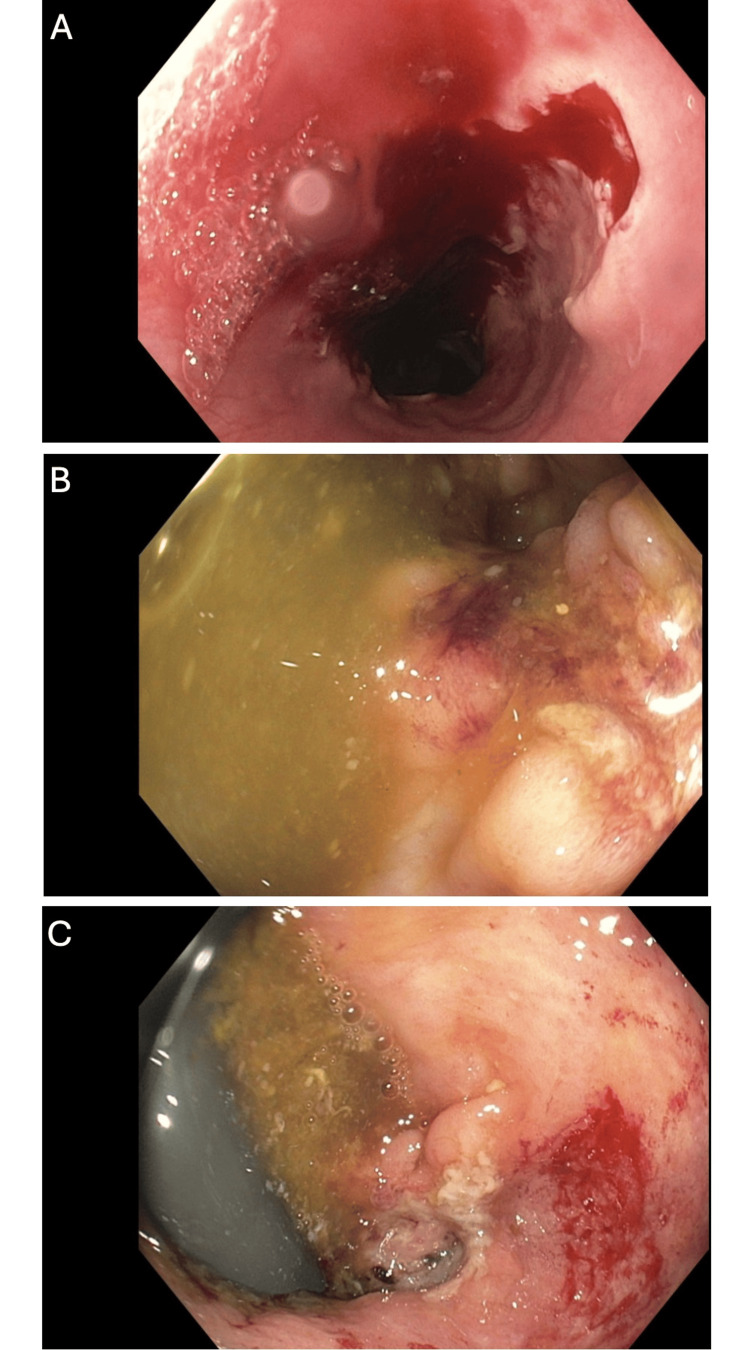
Endoscopy images demonstrating multiple ulcers in the distal esophagus (A), sigmoid colon (B), and rectum (C).

**Figure 4:**
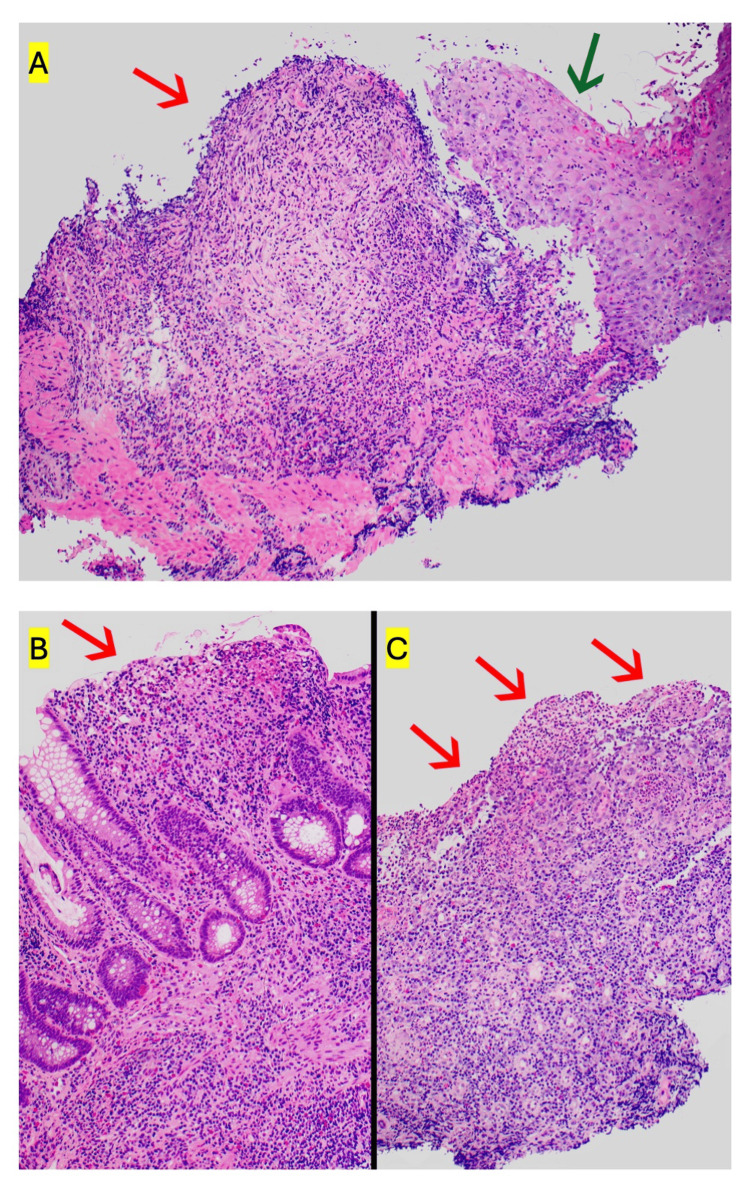
Hematoxylin and eosin stain from the (A) esophagus demonstrating ulceration with focal loss of mucosa (red arrow), as well as marked acute inflammation of remaining epithelium (green arrow). Histology from the colon demonstrating (B) focal erosion of inflamed glandular epithelium and (C) frank ulceration of the colon.

A 27-year-old female with a history of iron deficiency anemia presented to the emergency department with a two-week history of rapidly expanding bilateral lower extremity wounds. No history of overt bleeding was reported. On examination, multiple ulcers with undermined borders and a cribiform appearance were noted (Figure 1). Laboratory evaluation was notable for a white blood cell count of 6.90 x10^3^/uL, hemoglobin of 6.9 g/dL with a mean corpuscular volume of 66.2 fL, serum iron of 14 mcg/dL, ferritin of 3 ng/mL, and iron saturation of 3%. Erythrocyte sedimentation rate was 99 mm/hr, and C-reactive protein was 1.2 mg/dL (Table 1). Magnetic resonance imaging of the abdomen did not demonstrate any acute abnormalities. A punch biopsy of the wound was performed, and pathology demonstrated mid dermal mixed inflammation with abscess formation (Figure 2). Infectious stains were negative for pathogenic organisms, and tissue cultures were negative. Upper endoscopy and sigmoidoscopy were performed, demonstrating multiple esophageal, descending colon, sigmoid colon, and rectal ulcers (Figure 3). Pathology demonstrated severe acute esophagitis with ulceration and chronic colitis with ulceration (Figure 4). A diagnosis of Crohn’s disease with associated PG was made. The patient was initiated on infliximab, with subsequent resolution of the lower extremity ulcers and iron deficiency anemia.

**Table 1:** Comprehensive laboratory values on the day of hospital admission.

Test	Value	Reference range
Complete blood count		
White blood cell count	6.90 ×10³/µL	4.0-11.0×10³/µL
Hemoglobin	6.9 g/dL	Female: 12.0-15.5 g/dL
Hematocrit	26%	34.9-45.2 %
Mean corpuscular volume	66.2 fL	79.3-98.6 fL
Platelet count	369 ×10³/µL	150-400×10³/µL
Iron	14 mcg/dL	41-179 mcg/dL
Iron binding capacity	411 mcg/dL	262-502 mcg/dL
Iron percent saturation	3%	-
Ferritin	3 ng/mL	8-180 ng/mL
Erythrocyte sedimentation rate	99 mm/hr	<=25 mm/hr
C-reactive protein	1.2 mg/dL	<0.8 mg/dL
Metabolic panel		
Sodium	141 mmol/L	135-146 mmol/L
Chloride	2.9 mmol/L	3.6-5.3 mmol/L
Potassium	116 mmol/L	96-106 mmol/L
Bicarbonate	15 mmol/L	20-30 mmol/L
Urea nitrogen	4 mg/dL	7-22 mg/dL
Creatinine	0.29 mg/dL	0.60-1.30 mg/dL
Glucose	75 mg/dL	65-99 mg/dL

## Discussion

In this report, we describe a case in which PG was the presenting symptom leading to a diagnosis of IBD. This is unusual on several fronts: firstly, as described previously, PG is a rare condition, with an estimated prevalence between 5.6 and 6.1 cases per 100,000 adults in the United States [2]. Secondly, the diagnosis of PG often proves elusive as its clinical and histopathologic findings are nonspecific; indeed, PG is considered a diagnosis of exclusion [5].

Clinically, a PG lesion develops initially as a painful, tender nodule or pustule, which rapidly enlarges into an ulcer with undermined borders a purulent or necrotic base. As the lesions heal, they develop a cribiform appearance [1]. Histopathology demonstrates a mixed inflammatory cell infiltrate and abscess formation with epidermal and superficial dermal necrosis [5]. Although these histopathologic findings are nonspecific, biopsy remains crucial to exclude alternate etiologies including infection, malignancy, and cutaneous vasculitis [6]. In our case, the patient had initially been diagnosed with a purulent cellulitis but had not experienced clinical improvement despite several courses of antibiotics; ultimately, the histopathology did not demonstrate evidence of infection on tissue stains or culture.

The association between PG and IBD has been well established; in various reports, 34-41% of patients with PG were diagnosed with comorbid IBD [3,4]. Other diseases associated with PG include inflammatory arthritis, malignancy, and hematologic conditions such as myelodysplastic syndrome [4]. Although the pathogenesis of PG is not fully understood, it is likely that PG and its associated systemic conditions involve dysregulation of common immune pathways. Skin samples from PG lesions demonstrate significantly increased expression of interleukin (IL)-1β, IL-17, and tumor necrosis factor (TNF)-α, among other inflammatory cytokines [7,8]. Consequently, immunosuppressive therapies including TNF-α inhibitors, such as infliximab, have demonstrated clinical efficacy for both PG and IBD [9,10].

The treatment of PG is often multimodal and involves immunosuppression, wound care, and treatment of associated inflammatory conditions. For limited disease, topical corticosteroids may be considered, but high-dose systemic corticosteroids are the preferred initial therapy for patients with more extensive disease [1,3,11]. Rapid resolution of lesions may be seen upon initiation of corticosteroid therapy, with reports of complete resolution of skin lesions within weeks of therapy initiation [11]. In cases in which PG is comorbid with other inflammatory conditions, alternate agents may be preferred; for instance, in one cohort of 13 patients with refractory PG and IBD, initiation of infliximab resulted in complete healing of skin lesions in all patients [10].

## Conclusions

PG is a rare inflammatory skin disease that is frequently misdiagnosed, as it is a diagnosis of exclusion with nonspecific clinical and histopathologic findings. PG is highly associated with systemic inflammatory conditions such as IBD. Thus, when PG is suspected or confirmed in the absence of a known diagnosis of a systemic inflammatory condition, a broad work-up must be initiated to evaluate for an associated diagnosis.
